# Partial Rescue of Ocular Pigment Cells and Structure by Inducible Ectopic Expression of Mitf-M in MITF-Deficient Mice

**DOI:** 10.1167/iovs.18-25186

**Published:** 2018-12

**Authors:** Helen T. Michael, Cari Graff-Cherry, Sung Chin, Corinne Rauck, Amelework D. Habtemichael, Patricia Bunda, Tunde Smith, Maria M. Campos, Kapil Bharti, Heinz Arnheiter, Glenn Merlino, Chi-Ping Day

**Affiliations:** 1 Laboratory of Cancer Biology and Genetics, National Cancer Institute, National Insitutes of Health, Bethesda, Maryland, United States; 2 Laboratory Animal Science Program, National Frederick Laboratory for Cancer Research, National Insitutes of Health, Frederick, Maryland, United States; 3 Histopathology Core Facility, National Eye Institute, National Insitutes of Health, Bethesda, Maryland, United States; 4 Unit on Ocular and Stem Cell Translational Research, National Eye Institute, National Insitutes of Health, Bethesda, Maryland, United States; 5 Scientist Emeritus, National Institute of Neurological Disorders and Stroke, National Institutes of Health, Bethesda, Maryland, United States

**Keywords:** melanocytes, retinal pigment epithelium, microphthalmia

## Abstract

**Purpose:**

Complete deficiency of microphthalmia transcription factor (MITF) in *Mitf^mi-vga9/mi-vga9^* mice is associated with microphthalmia, retinal dysplasia, and albinism. We investigated the ability of dopachrome tautomerase (DCT) promoter-mediated inducible ectopic expression of Mitf-M to rescue these phenotypic abnormalities.

**Methods:**

A new mouse line was created with doxycycline-inducible ectopic *Mitf-M* expression on an Mitf-deficient *Mitf^mi-vga9^* background (DMV mouse). Adult DMV mice were phenotypically characterized and tissues were collected for histology, immunohistochemistry, and evaluation of *Mitf*, pigmentary genes, and retinal pigment epithelium (RPE) gene expression.

**Results:**

Ectopic *Mitf-M* expression was specifically induced in the eyes, but was not detected in the skin of DMV mice. Inducible expression of *Mitf-M* partially rescued the microphthalmia, RPE structure, and pigmentation as well as a subset of the choroidal and iris melanocytes but not cutaneous melanocytes. RPE function and vision were not restored in the DMV mice.

**Conclusions:**

Ectopic expression of *Mitf-M* during development of Mitf-deficient mice is capable of partially rescuing ocular and retinal structures and uveal melanocytes. These findings provide novel information about the roles of *Mitf* isoforms in the development of mouse eyes.

Although all melanin-bearing pigment cells of vertebrates come from the neuroectoderm, they can be divided into two principally distinct classes. The retinal pigment epithelium (RPE) cells are derived from the neuroepithelium of the ventral forebrain, and the melanocytes in skin and its appendages and various extracutaneous locations are derived from the neural crest. Nevertheless, the development of both neuroepithelium- and neural crest–derived melanocytes depends on the same gene, microphthalmia-associated transcription factor (*Mitf*), which encodes a set of distinct isoforms of a helix-loop-helix-leucine zipper transcription factor collectively called MITF proteins.^1^ During mouse development, *Mitf* is first expressed around embryonic day 9.5 in precursors of the pigment cells, soon followed by the expression of a gene whose protein product is later involved in melanogenesis, dopachrome tautomerase (*Dct*).^2^ In the RPE, *Mitf* expression reaches its peak during the following embryonic days but then is progressively reduced.^3,4^ Among the neural crest–derived cells, *Mitf* expression marks the melanocyte precursors called melanoblasts, and *Mitf* continues to be expressed in their melanocytic derivatives along with *Dct*, particularly in skin and feather and hair follicles.^2^

MITF has many functions in melanocyte development and maintenance, chief of which are the regulation of cell specification, proliferation, and differentiation. In the total absence of functional Mitf, as seen in mice homozygous for the *Mitf*^mi-vga9^ allele, mice exhibit microphthalmia and have neither pigmented RPE cells nor pigmented neural crest–derived cells in skin, iris, choroid, inner ear, or heart.^3^ Nevertheless, there is a fundamental difference in the way these two types of pigment cells respond to *Mitf* loss-of-function mutations. Neural crest–derived, *Dct*-expressing melanoblasts can be seen for only a short time before they disappear.^3^ In contrast, in the presumptive RPE, the mutant cells hyperproliferate and continue to express *Dct*.^3^

These findings prompted us to test whether inducible ectopic expression of *Mitf*, if achieved early enough during development and at sufficient levels, might rescue the pigment cells in question, and whether later removal of *Mitf* might affect the cells once formed. To achieve ectopic expression, we prepared a line of transgenic mice modeled after our previously described transgenic mice in which a Dct promoter–driven reverse tetracycline–controlled transactivator (Dct-rtTA) activates GFP expression under the control of a tetracycline-responsive element (TRE).^5^ This system allows for targeting GFP expression to both neural crest–derived melanoblasts/melanocytes and RPE cells during all stages of development and in adulthood.^5^ We anticipated that a similar targeted expression might be achieved for *Mitf*.

## Methods

### Derivation of DMV Mice

We produced a transgene construct, TRE-Mitf-M-V5 (Mi-V5), in which a cDNA of mouse *Mitf-M* (419 residue; referred as [+] isoform) with a V5 tag sequence at its carboxyl end (Mi-V5) was placed under the control of a TRE (Supplementary Fig. S1A). The Mi-V5 transgene construct was injected into zygotes heterozygous for *Mitf^mi-vga9^* and Dct-rTA obtained by appropriate crosses and eventually generated a bi-transgenic line of mice on an *Mitf*-deficient background (Supplementary Fig. S1B). This line of mice is hereafter called DMV, and the littermate without the *Mitf* transgene DV. All mouse experiments were conducted following National Institutes of Health guidelines for the care and maintenance of mice and ARVO Statement for the Use of Animals in Ophthalmic and Vision Research. Experiments were approved by the Animal Care and Use Committee at National Cancer Institute Frederick. All experiments were performed on mice older than 1 month.

### Reporter Assay

MDCK Tet-Off cells were co-transfected with the TRE-Mi vector, the Mitf-responsive reporter E3-hTRPM1-pGL3 (a gift from David Fisher, Massachusetts General Hospital, Boston, MA, USA),^6^ and the normalizer reporter RL vector (#E2231; Promega, Fitchburg, WI, USA) in a 5:1:0.2 ratio using FuGene HD (#E2311; Promega), following the manufacturer's instructions. Twenty-four hours after transfection, cells were treated overnight with either 2 μg/mL doxycycline (dox) or vehicle. The cells were lysed for use in a dual-luciferase assay (Dual-Luciferase Reporter Assay System, #E1910; Promega), following the manufacturer's instructions.

### Western Blot

Mouse ES cell line (a gift from Lino Tessarollo, Mouse Cancer Genetics Program; National Cancer Institute, Frederick, MD, USA) was transfected with the TRE-Mi-V5 vector, and a stable transfectant clone (ES/TRE-Mi) was selected by hygromycin treatment for 2 to 4 weeks. A pTet-On vector (#631018; Takara Biosciences, Mountain View, CA, USA) was transfected into the ES/TRE-Mi cells using FuGene HD (see above) for 24 hours. Cells were then treated overnight with 2 μg/mL of dox or vehicle and harvested for Western blot using anti-MITF antibody (a gift from David Fisher)^7^ and HRP-conjugated V5 Tag Monoclonal Antibody (# R961-25; ThermoFisher Scientific, Waltham, MA, USA).

### Mouse Genotyping

Genomic DNA (gDNA) was prepared for genotyping from mouse tail clips by the HotSHOT protocol.^8^ In brief, mouse tail clips, 2- to 3-mm long, were immersed in 75 μL of alkaline lysis buffer (25 mM of NaOH and 0.2 mM of EDTA, pH = 12) and heated at 95°C for 30 to 40 minutes. After cooling on ice for 1 minute, 75 μL of neutralization buffer (40 mM of Tris-HCl, pH = 5) were added. The PCR reaction contained 1× GoTaq Green Mix (No. M712; Promega), primers (0.5 μM each), gDNA from HotShot method (2 μL), and water for a final volume of 25 μL (Supplementary Table S1).

The genotyping of the *Mitf*^mi-vga9^ (vga9) allele requires two PCR reactions, Mi-UP and LacZ. Homozygous vga9 yields LacZ-positive, no-Mi-UP results. Heterozygous vga9 yields both LacZ and Mi-UP positive results. Wild-type *Mitf* yields no-LacZ, but Mi-UP–positive results. The genotyping PCRs for Dct-rtTA and TRE-Mi-V5 use primers of rtTA and TRE-Mi, respectively.

### Reverse Transcription–Polymerase Chain Reaction

The tissues harvested from mice were flash frozen in liquid nitrogen and stored at −80°C. To prepare RNA, the frozen tissue was pulverized using Cryoprep System (#CP-01; Covaris, city, state, country), and the powder was lysed for RNA preparation following the instruction of RNeasy Mini Kit (#74106; Qiagen, Germantown, MD, USA). cDNA was synthesized using Invitrogen SuperScript III First-Strand Synthesis System (#18080051; ThermoFisher Scientific). The PCR reaction mixture was prepared as mentioned in the Mouse genotyping section. The primers and thermocycles are described in Supplementary Table S2.

### Scoring of Eye Size

Eye size was scored as “micro,” “medium,” or “large” based on photographs of adult mice taken by CS and CGC. Mice were then classified by genotype and dox treatment. Eyes that could not clearly be seen in the photographs were excluded from evaluation. Classification was performed for 136 eyes from DV mice, 43 eyes from DMV −dox mice, 83 eyes from DMV +dox mice treated with 2 g/kg dox and 35 eyes from DMV +dox mice treated with 0.2 g/kg dox. A X^2^ test was performed to analyze the distribution using Prism version 7 (Graphpad Software, La Jolla, CA, USA).

### Histology and Immunohistochemistry

Tissues were fixed in 10% neutral buffered formalin for 24 hours and then moved to 70% ethanol until being sent to Histoserv, Inc. (Gaithersburg, MD, USA) for paraffin embedding, sectioning, and hematoxylin and eosin (H&E) staining. The immunohistochemistry protocol was performed as published^9^ with the addition of hydrogen peroxide melanin bleaching following antigen retrieval. Briefly, slides were deparaffinized in xylene and rehydrated through graded alcohols. Antigen retrieval was performed using Target Retrieval Buffer pH 6 (#S1699; Dako, Santa Clara, CA, USA) with steam (Farberware Programmable Pressure Cooker; Farberware, Vallejo, CA, USA) for 10 minutes followed by further incubation for 10 minutes on the benchtop. For melanin bleaching, 10% hydrogen peroxide was heated to 60°C, slides were incubated at 60°C in warm H_2_O_2_ for 10 minutes and then washed in TBS for 5 minutes. After addition of a nonspecific protein blocker (X0909; Dako), slides were stained with a Dct antibody (Pep8h, gift from Vincent Hearing, National Cancer Institute) by incubation at a 1:5000 dilution overnight at 4°C. The staining was revealed using ImmPRESS anti-rabbit alkaline phosphatase polymer (Vector Laboratories, Burlingame, CA, USA), developed with Vector Red chromogen (Vector Laboratories) followed by counterstaining with Mayer's hematoxylin (#MHS32; Sigma Aldrich, St. Louis, MO, USA) before coverslipping.

### Ocular Development

Mouse fetuses were collected at 10.5, 13.5 and 14.5 days of gestation and put into 4% paraformaldehyde for 1 hour (10.5 days) or 2 hours (13.5 and 14.5 days) and then moved to graded sucrose solutions for dehydrations. Images of the entire fetus and the developing optic cup were taken for 14.5-day fetuses using a camera and the EVOS Cell Imaging system (ThermoFisher Scientific). Fixed fetuses were frozen in optimum cutting temperature (OCT) medium (Sakura Finetek, Torrance, CA, USA) and coronal sections were stained. Briefly, coronal sections were blocked with 10% normal goat serum for 30 minutes, followed by overnight co-incubation at room temperature with anti-MITF (C5 Ab12039 1:1000; Abcam, Cambridge, MA, USA) and anti-V5 antibody (Abcam Ab9116 1:300). Samples were then incubated with secondary goat anti-mouse 488 (1:300), goat anti-rabbit 555 (1:300; ThermoFisher Scientific), and DAPI 405 (1:2000; ThermoFisher Scientific) in ICC Buffer for 30 minutes (ThermoFisher Scientific). Immunolabeling was imaged using a LSM 800 confocal microscope (Zeiss, Thornwood, NY, USA).

## Results

### Ectopic Expression of Mitf-M in MITF-Deficient Mice During Embryogenesis Rescued Ocular Size, But Not Coat Pigmentation

**Figure 1:**
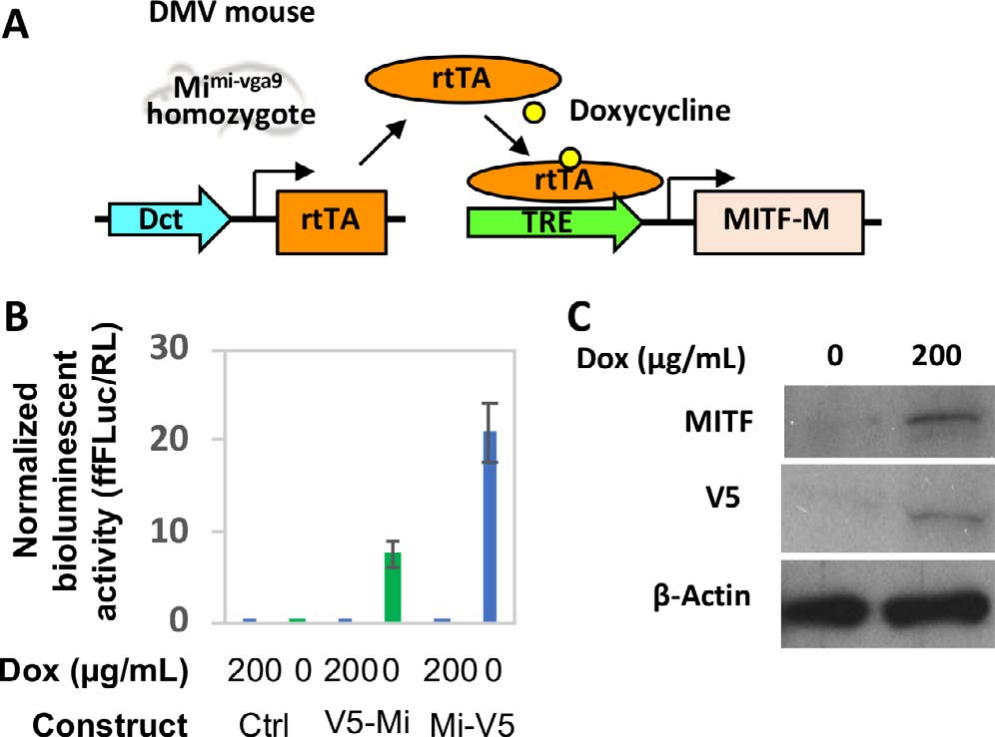
Generation of DMV mice and function of Mi-V5 transgene construct. (A) Design of Dct-rtTA/TRE-Mitf-M-V5/Mitf^mi-vga9^ homozygous (DMV) mice that allows induction of the Mitf-M-V5 transgene on an Mitf-null background. (B) Two TRE promoter-driven Mitf-M constructs were tested. One had a V5 tag on the amino terminal end of the Mitf-M cDNA (V5-Mi) and the other a V5 tag on the carboxy terminal end (Mi-V5). MDCK cells with a Tet-Off rtTA construct were co-transfected with the Mitf-responsive reporter (E3-hTRPM1-pGL3) and normalizer reporter (RL vector) for dual luciferase, and also with the empty vector controls, V5-Mi or Mi-V5 vector. Twenty-four hours after transfection, cells were treated with either 200 μg/mL dox or vehicle overnight and luciferase was measured. Mi-V5 showed higher Mitf activation than the V5-Mi construct. (C) Mouse ES cells with a Tet-On vector were stably transfected with the TRE-Mi-V5 vector and cloned from single cells. Cells were treated overnight with 200 μg/mL of dox or vehicle and harvested for Western blot analysis. Dox-treated cells showed both Mitf and V5 protein expression.

**Figure 2:**
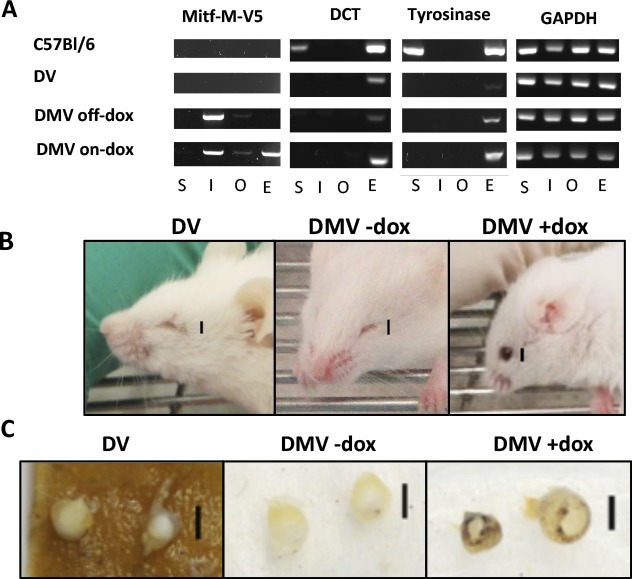
Expression of Mitf and other pigmentary pathway genes in adult DMV mice. (A) Gene expression patterns as assayed by RT-PCR showed that dox treatment induced expression of the Mitf-M-V5 transgene, as well as target genes Dct and Tyr in eyes (E) of DMV mice as compared with eyes of untreated DV and DMV mice. Minor leakiness of the transgene was observed in the intestine (I) and olfactory bulb (O). Expression of these genes is only seen in skin (S) of wild-type mice. (B, C) Continuous dox treatment throughout embryogenesis partially rescued the microphthalmia and pigmentation in a DMV mouse (DMV +dox), in comparison with a noninduced DMV mouse (DMV −dox) and a mouse without the Mi-V5 transgene (DV). Images of the representative mice (B) and globes (C) are seen in the upper and lower panels, respectively. Scale bars for mouse images represent 3 mm and scale bars for globes represent 2 mm.

One of the most abundant isoforms of MITF in neural crest–derived melanocytes is MITF-M while in the RPE other isoforms (MITF-A, MITF-H, MITF-D) are more abundant,^10–12^ although low levels of MITF-M can also be found, at least in adult human and bovine RPE.^13^ As available evidence suggests that the different isoforms do not regulate vastly different sets of target genes, we reasoned that dox-inducible ectopic expression of MITF-M in *Mitf^mi-vga9^* homozygotes might be able to rescue not only neural crest–derived melanocytes but RPE cells as well (Fig. 1A). The function and inducible expression of the Mi-V5 construct was confirmed using an Mitf-responsive reporter assay and Western blotting in cell lines expressing tetracycline-controlled transactivators (Figs. 1B, 1C). The Mi-V5 construct was then used to derive the DV and DMV mice on an *Mitf*-deficient *Mitf^mi-vga9^* background (Supplementary Figs. S1A, S1B). As anticipated, when mothers carrying DMV embryos were treated with dox, transgenic *Mitf-M* could be induced in the offspring (Fig. 2A). During embryogenesis, pigment was grossly visible in the optic cup of DMV +dox embryos by 13.5/14.5 days of development but not in DV littermates (Supplementary Fig. S2A) or in 10.5-day embryos. Using ex vivo imaging, individual pigment cells were visible in the developing optic cup of 13.5-day DMV +dox embryos but were absent from DV eyes (Supplementary Figs. S2B, S2C). Pigmented cells are present in the developing RPE and choroid (Supplementary Fig. S2D). *Mitf* transgene expression was faintly detectable in treated 10.5-day embryos (Supplementary Figs. S3A, S3B). By 13.5 days, immunolabeling of the V5 tag was demonstrated in the developing RPE layer. Some nonspecific immunolabeling is also seen with the anti-Mouse 488, showing some nonspecific staining (Supplementary Fig. S3D, inset). When the dox-treated DMV pups were allowed to grow to adulthood, their coat remained as white as that of untreated DMV controls or transgene-negative *Mitf^mi-vga9^* homozygotes. Their eyes, however, were considerably larger and visibly pigmented compared with those of untreated controls (Fig. 2B). Among the organs tested, *Mitf-M* induction was dependent on dox and efficient in the eye. Nevertheless, while dox-dependent in the eye, transgene expression was leaky in the intestine and the olfactory bulb. Interestingly, we never detected induction in the skin.

**Figure 3:**
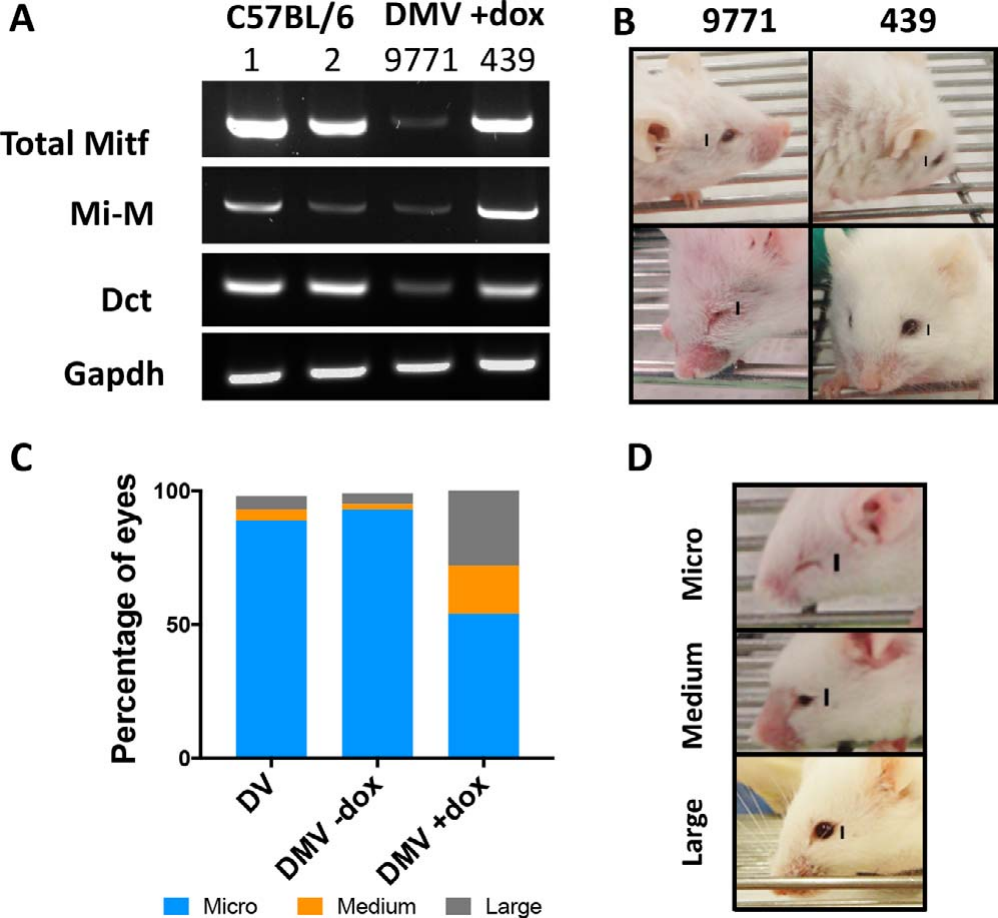
Ocular phenotypes in adult DMV mice. (A) RT-PCR results indicated that total Mitf and Mitf-M expression varies in eyes from different DMV mice. Total Mitf expression in the eyes of DMV mouse #439 was similar to control C57BL/6 mice. Both total and Mitf-M expression was higher in eyes from mouse #439 than from mouse #9771. (B) Rescue of ocular structure and size varied in individual DMV mice. Mouse #9771 has only has one eye open while #439 had both eyes open and larger eye size. (C) Although size variation was seen in the eyes of all groups, the percentage of medium and large eyes was significantly higher in the DMV +dox group than in the DV or DMV −dox group. Classification was performed for 136 DV eyes, 43 DMV −dox eyes, and 118 DMV +dox eyes. X^2^ test for distribution was performed and showed significant difference between columns (P < 0.0001). (D) Representative examples of eyes classified as micro, medium, or large. Scale bars for mice represent 3 mm.

*Mitf-M* and total *Mitf* expression were similar in control C57BL/6 and DMV +dox eyes, as was the expression of *Dct*, although there were individual variations in expression levels (Fig. 3A). Variation in eye size in DMV +dox mice was often related to the level of transgene expression (Figs. 3A, 3B). Large eyes were in general significantly (*P* < 0.0001) more common in DMV +dox mice (28%) than in DMV −dox (5%) or DV mice (4%) (Figs. 3C, 3D). This variability was reduced after treatment with 2 g of dox/kg as opposed to 0.2 g of dox/kg (Supplementary Fig. S4). Rarely, large eyes were also seen in the DV population, perhaps due to genetic variability introduced from an initial C3H outcross arranged to increase fertility when establishing the transgenic line (Supplementary Fig. S5). Pigmented hairs, however, were never found on any of the DMV pups or adults, perhaps because the ectopic *Mitf-M* expression started too late in embryogenesis, remained at too low levels, or lasted for too short a time period to rescue melanoblasts. From these results, we concluded that Mi-V5 was inducible and functional in DMV mice but only in their eyes.

### Mitf-M Expression Partially Restored Retinal Structure but Not Function

**Figure 4:**
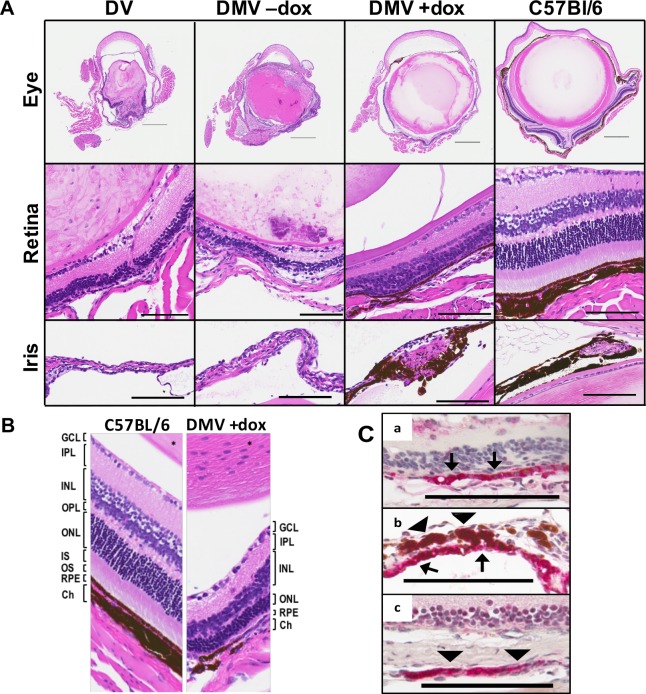
Partial rescue of eye structure in adult dox-treated DMV mice. (A) H&E staining showed the structures of eye globes, retina, and iris of the adult mice, as indicated. DMV mouse eyes exhibit a rescue of globe size, retinal layers, and iris and choroidal pigmentation. (B) The retina is not fully rescued in the DMV +dox mouse. The photoreceptor IS and OS layers are still absent in the DMV +dox mice. (C) Dct staining in the RPE (a, b) and in melanocytes in iris (b) and choroid (c) of a DMV +dox mouse. Ch, choroid. Arrows: RPE. Arrow heads: melanocytes. Scale bar: 200 μm (A, top row) or 100 μm (A, middle; and B, C, bottom row).

**Figure 5:**
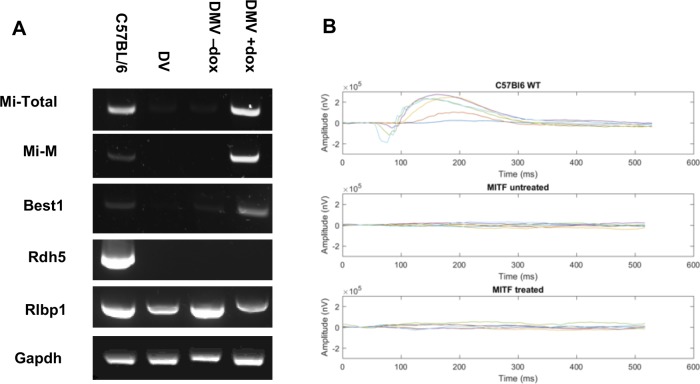
Inducible expression of Mitf-M rescued only one of three tested RPE genes. (A) The results of RT-PCR targeting total Mitf, Mitf-M (both including Mi-V5 transgene), and the RPE marker genes Best1, Rdh5, and Rlbp1. Gapdh is shown for control purposes. Note that the Mitf (Mi-total in DMV w/dox #439) was induced at levels comparable to those of wild-type C57BL/6 eyes, and Mitf-M (Mi-M, transgene + endogenous Mitf-M) at even higher levels than in the wild-type control. Of the RPE genes, only Best1 is induced upon dox treatment, while Rdh5 is not, and Rlbp1 expression is not affected by the presence of Mitf. (B) Electroretinograms showed lack of VER in DMV mice +dox or −dox (middle and lower panel, respectively), as compared with the VER of wild-type mice (upper panel). All results shown are from adult mice.

The above findings were intriguing and prompted us to ask whether pigment cell rescue in the eye was restricted to the RPE or involved other eye structures as well, in particular also neural crest–derived iris and choroidal cells. In addition to microphthalmia, *Mitf^mi-vga9^* mice have structural abnormalities in the eyes, including RPE hyperplasia and loss of normal retinal architecture.^3,14–16^ The histologic analysis of adult eyes (Fig. 4), indicated not only an increase in size but also an overall improvement of the anatomy of the eye, showing a more easily identifiable and less dysplastic lens, and pigmented choroidal and iris melanocytes, either isolated or clustered in small groups. These latter cells were also positive for DCT, consistent with the suggestion that they represent true melanocytes (Fig. 4C). Nevertheless, rescue of these cells is incomplete and estimated not to exceed 5% compared with control. Dox treatment also led to a more organized RPE, with a number of its cells pigmented, in contrast to untreated controls, whose RPE remained unpigmented and hyperplastic (Figs. 4A middle row, 4B, 4Ca, 4Cb). Among the various improvements in eye anatomy we also saw an improvement of the layered structure of the retina. While in untreated controls, the outer nuclear layer (ONL), outer plexiform layer (OPL), inner nuclear layer (INL), and inner plexiform layer (IPL) are all thinned out (Fig. 4A, middle row), these layers are by comparison thicker in the treated DMV mice (Fig. 4A, middle row, and 4B). Nevertheless, photoreceptor outer and inner segments (OS and IS) remain absent regardless of treatment (Fig. 4B). Improvement in the RPE was additionally supported by expression of *Best1*, an RPE marker, in rescued RPE but not controls (Fig. 5A). However, expression of the retinol dehydrogenase *Rdh5*, which participates in the regeneration of 1-cis-retinal required for photoreceptor function, was not rescued (Fig. 5A).^16^ We also tested whether the rescued eyes would respond to visual stimuli by measuring their visual-evoked response (VER) in electroretinograms. Not surprisingly, they did not (Fig. 5B). This finding is consistent with previous work showing that the RPE is integral for normal photoreceptor layer development.^17^ Lastly, we asked whether later dox removal would have any effect on the pigmented cells. Hence, we discontinued treatment in two mice at 33 days and one mouse at 72 days after birth but saw no significant gross or microscopic changes in the eyes for up to 90 days thereafter, suggesting that MITF-M expression is not required for maintenance of ocular elements.

## Discussion

Expression of *Mitf-M* under the control of a Dct promoter in *Mitf*-null mutant mice was able to partially rescue the structure of the eye, though not its visual function, and along with it a fraction of the RPE cells and choroidal and iris melanocytes, although the neural crest origin of these latter rescued cells still remains to be confirmed. No rescue, however, was achieved for cutaneous melanocytes. This was similar to a previous study showing a partial rescue of ocular pigmentation but not hair pigmentation using a tyrosinase-rtTA transgene in albino mice.^18^ We posit that the failure to rescue skin melanocytes and variable rescue of ocular pigment cells could be due to either toxicity of the rtTA system, missing a critical window of MITF expression before DCT expression arises, and/or insufficient MITF expression driven by the transgenic Dct promoter. Although toxicity of rtTAs have been reported,^19^ Dct-rtTA LacZ animals show rtTA expression in both eyes and cutaneous melanocytes^20^ and expression of the transgenes throughout development is routine in our laboratory for visualizing cutaneous melanocytes. It is possible, however, that the period in normal development between the first onset of MITF-M expression and subsequent DCT expression is critical for survival of pigment cell precursors in the skin. Because the use of Dct-rtTA would miss such a window, this would account for failure to rescue cutaneous melanocytes. Additionally, the dose of MITF-M may have been insufficient to rescue skin melanocytes and fully rescue the RPE. Although our Dct-rtTA TRE-H2BGFP system successfully results in expression of GFP in melanocytes, various causes, including integration site position of the transgene, strain background, dose responses, and epigenetic silencing, have been suggested for variegated or insufficient transgene expression in mice.^18,21–24^ Although DMV mice have near normal MITF expression in the eyes in adult mice, we unfortunately cannot assess expression in cutaneous melanocytes because failure of expression (for any reason) necessarily results in an absence of skin melanocytes. Previous work has shown that expression of inducible GFP with this Dct-rtTA is higher when both transgenes are homozygous as opposed to heterozygous.^5^ Combined with the better rescue observed at the higher dox dose, this suggests that homozygous Dct-rtTA Mi-V5 mice may show a more complete rescue of ocular structures and pigment cells.

With respect to eye development, our findings confirm the critical role of RPE in retina development and raise interesting questions concerning the functional role of distinct MITF isoforms. Previous results have shown that selective nonconditional knockouts of *Mitf-D* or conditional suppression of *Mitf-D* by Dct-Cre–mediated knockout of *Pax6* led to shifts in the expression of other MITF isoforms but no significant changes in total *Mitf* expression and no visible perturbations in eye development.^14,25^ Moreover, the lack of MITF-M in mice homozygous for the extant allele *Mitf^mi-bw^*, although leading to the absence of choroidal melanocytes and melanocytes in the anterior layer of the iris, nevertheless leaves the RPE (and pigmentation of the posterior layer of the iris) as well as the overall structure of the eye intact.^26^ That MITF-M, normally absent in the developing RPE in mice, partially rescued *Mitf*-null mutant RPE is, however, consistent with the earlier observation that a close relative of MITF, TFEC, rescued the RPE of *Mitf^mi-rw^* homozygotes.^14^ Our findings suggest that Mitf-M +6aa isoform expression during development can rescue RPE hyperplasia in MITF-null mice, in contrast to recent reports that only the -6aa isoform suppressed proliferation in a human RPE cell line.^27^ The rescue of iris and choroidal as opposed to cutaneous melanocytes might indicate a different timing or level of expression of the rescue transgene in the different cell types, perhaps hinting at the earlier notion that distinct subpopulations of neural crest–derived melanocytes are not all created equal. In any event, our model for inducible expression of MITF may lend itself to future studies of the functional role of Mitf isoforms, *Mitf* relatives and other genes for the development of the various subpopulations of melanin-bearing pigment cells in mice.

## Supplementary Material

Supplement 1
