# Supplementary material for: Partial Rescue of Ocular Pigment Cells and Structure by Inducible Ectopic Expression of Mitf-M in MITF-Deficient Mice
Source: Invest Ophthalmol Vis Sci. 2018 Dec;59(15):6067–73. doi: 10.1167/iovs.18-25186 (PMC6314104; doi:10.1167/iovs.18-25186)
Supplement: Supplement 1 [file iovs-59-15-16_s01.pdf]

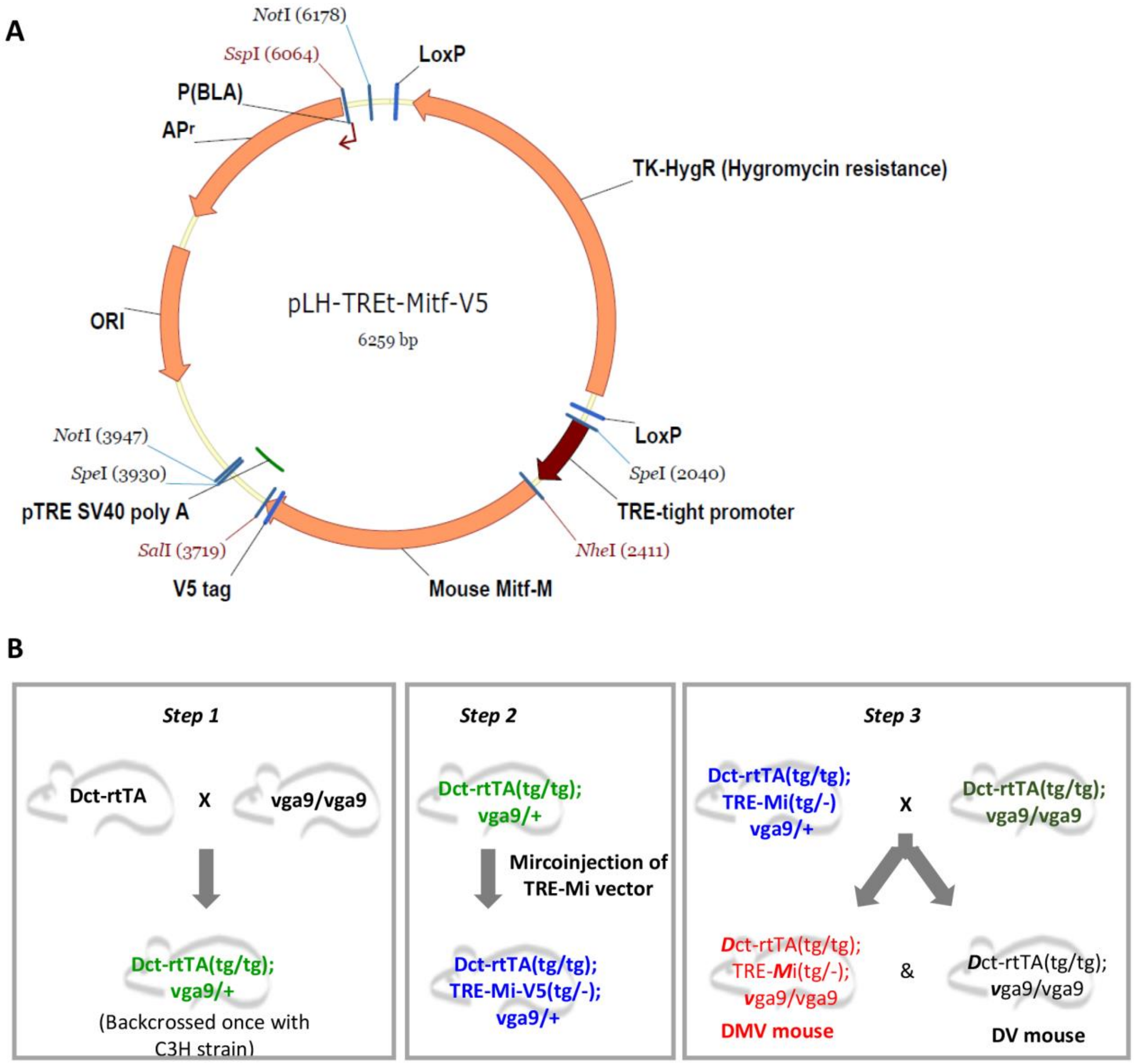

Supplementary Figure S1. Transgene design and generation of the inducible Mitf-M model.

A, Targeting vector containing the Mitf-M transgene (Mi-V5) used for the generation of DMV mice. **B,** Breeding scheme used to generate DMV mice. *Step1:*Dct-rtTA and *Mitf*$^{mi\text{-}vga9/mi\text{-}vga9}$ (labeled as vga9/vga9) mice on a C57BL/6 background were bred together to generate Dct-rtTA-homozygous, vga9-heterozygous (Dct-rtTA(tg/tg);vga9/+) mice. The mice were backcrossed with mice of the C3H strain for one generation to improve fertility, which might contribute to some of the observed phenotypic variability. All other mice used to generate the DMV line were

of the C57BL/6 strain. *Step 2:*The vector containing the transgene was microinjected into zygotes of the genotype (Dct-rtTA(tg/tg);vga9/+) to generate Dct-rtTA(tg/tg);TRE-Mi-V5(tg/-);vga9/+ mice. *Step 3:*Bi-transgenic Dct-rtTA(tg/tg);TRE-Mi-V5(tg/-);vga9/+ mice were bred with Dct-rtTA(tg/tg);vga9/vga9 mice. For Mi-V5 transgene induction, pregnant females were given doxycycline-containing chow (+dox group). Others were given regular chow (-dox group). The females gave birth to pups of the following genotypes: Dct-rtTA(tg/tg);TRE-Mi (tg/-);vga9/vga9 (DMV), Dct-rtTA(tg/tg);vga9/vga9 (DV), Dct-rtTA(tg/tg);TRE-Mi (tg/-);vga9/+, and Dct-rtTA(tg/tg);vga9/+ (the latter two types of pups are not shown in the figure). To generate and maintain a colony of DMV mice, DMV mice were bred with either DMV or DV mice.

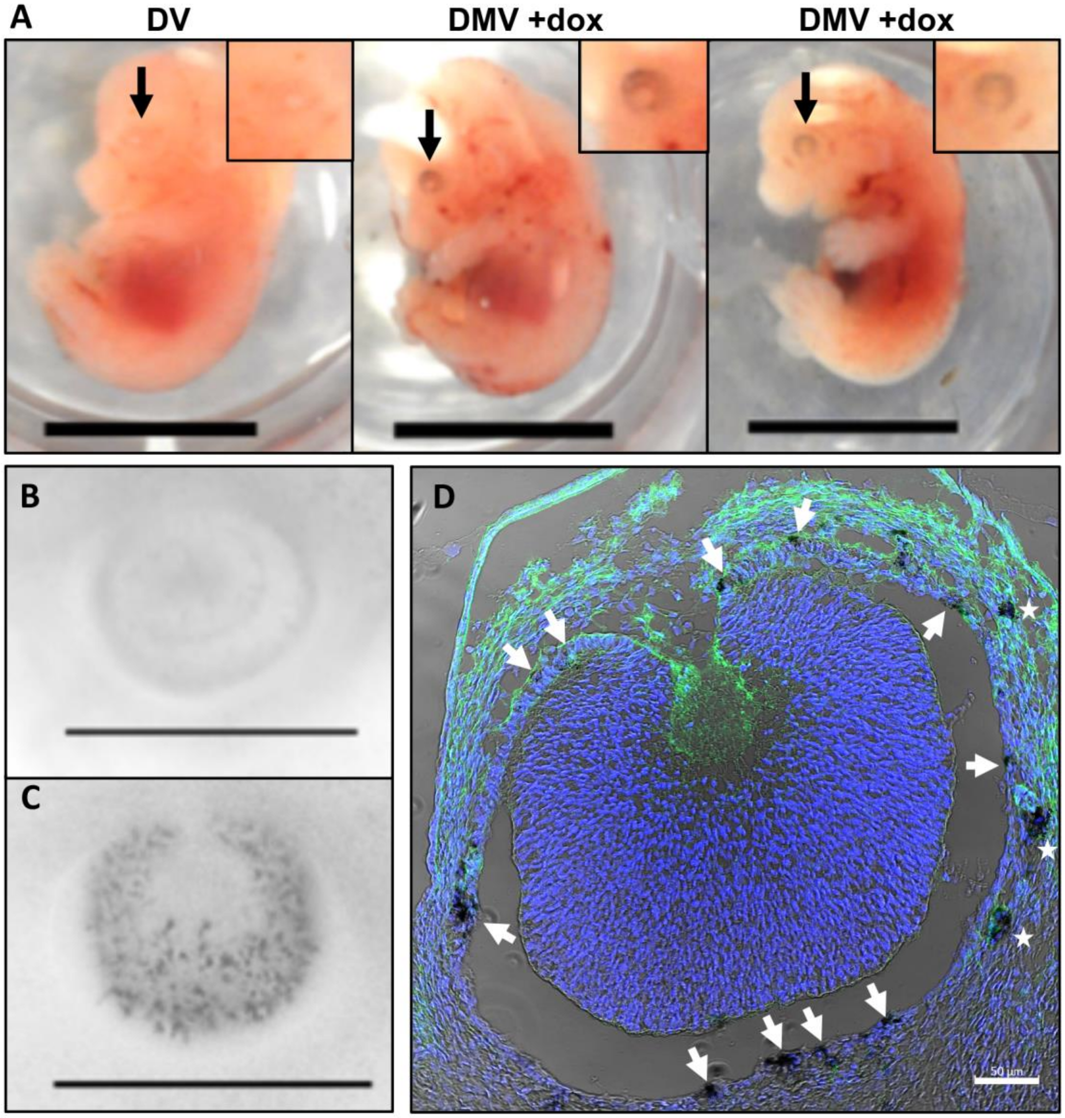

Figure S2. Partial pigmentation of the optic cup is seen in 13.5-14.5 day DMV +dox embryos. A, Pigmentation can be seen in the optic cup of DMV +dox 14.5 embryos but are absent in DV littermates (arrows and insets). **B and C,** Magnification (10x) of optic cup shows a lack of pigment in the DV embryo (B) compared with the DMV +dox embryo (C) that shows individual pigmented cells in the developing RPE (size bar = 1000µM). **D,** Photomicrograph of brightfield and fluorescence immunolabeling demonstrates pigmented cells in the developing RPE (white arrows) and occasionally outside of the RPE apparently in the developing choroid layer (white stars).

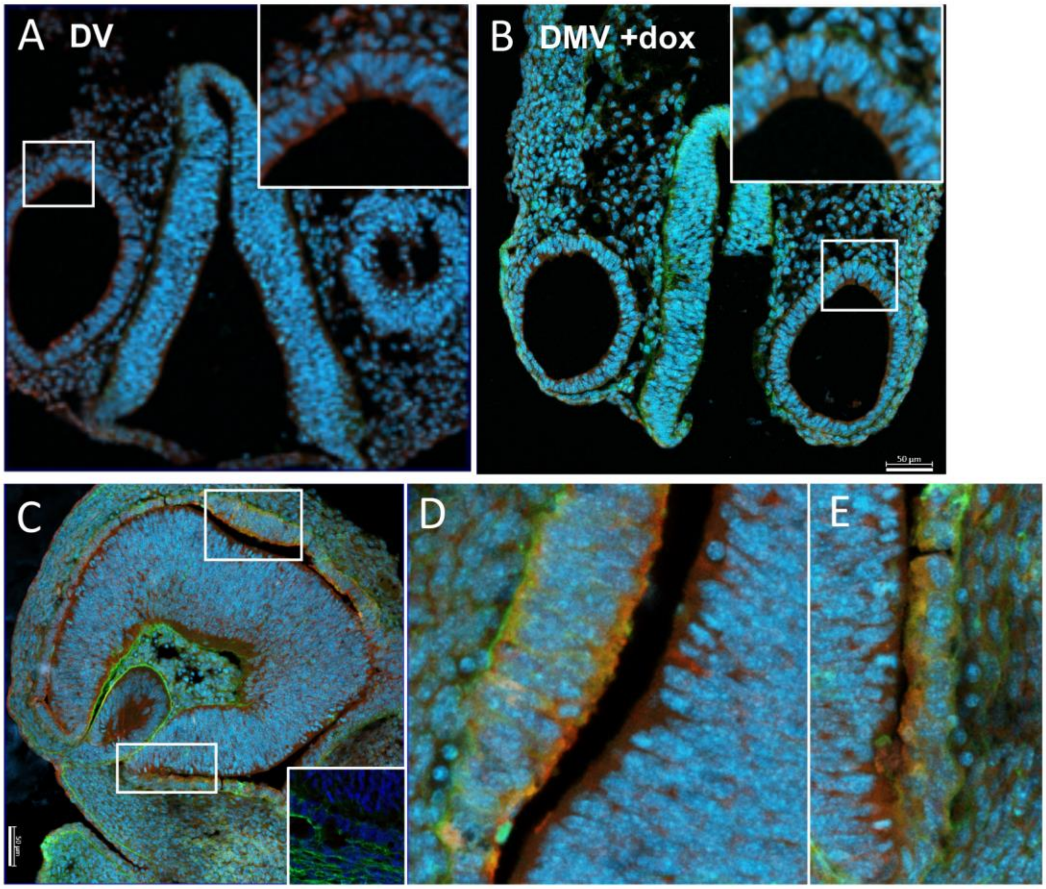

Figure S3. MITF expression seen starting as early as 10.5 days of gestation. A and B, Faint MITF immunolabeling (green) is seen in 10.5 day DMV +dox embryos but missing from DV littermates. Insets show magnification of area in white box. **C-E,** Anti-MITF (green) and Anti-V5 tag (red) immunolabeling is seen in 13.5 day embryo optic cup. Immunolabeling of the V5 tag was demonstrated in some areas along the developing RPE and adjacent neuroretina. Some non-specific immunolabeling with the 488 fluorophore was seen in negative control tissue (inset). **D** and **E** show magnification of area in white boxes. Size bars are 50μm.

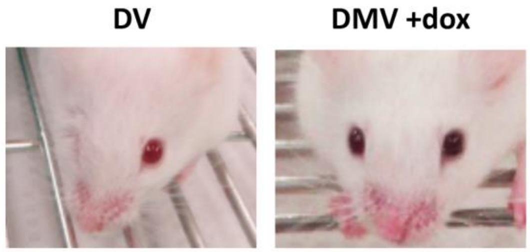

Figure S4. Comparison of pigmentation in eyes DMV +dox mice and DV mice. DVM +dox eyes have partial pigmentation, compared with DV eyes.

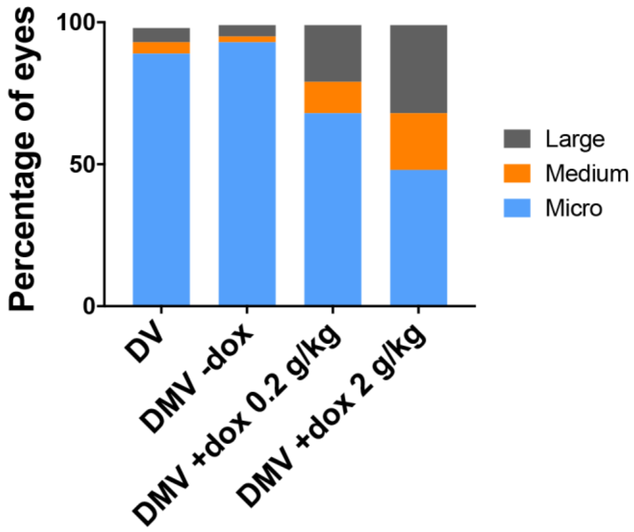

Figure S5. Rescue of the eye structure is dependent on the dose of doxycycline. The higher 2g doxycycline/kg dose has a higher percentage of medium and large eyes (20% and 31%, respectively) compared with the lower 0.2g doxycycline/kg dose (11% and 20%, respectively). D, Representative images are shown of eyes characterized as “micro,” “medium” and “large.”

Table S1. Primers and thermocycle program of genotyping PCR for DMV mice

Target	Forward and reverse primer	Size (bp)	Thermocycle
Mi-UP	5’-TGAAAGAGAAACACCATTGTCC-3’	450	(1) 94°C, 5min.<br>(2) 94°C, 1min.<br>(3) 55°C, 1min.<br>(4) 72°C, 3min.<br>Repeat (2)-(4) for 30 cycles.<br>(5) 72°C, 10min
	5’-AGCATTTCCAGCATAGCAAG-3’		
LacZ	5’-CGTCAGTATCCCCGTTTACAG-3’	120	
	5’-TATCGCCAAAATCACCGCC-3’		
rtTA	5’-ACTAAGTAAGGATCAATTCAG-3’	350	
	5’-TGTACTAGGCAGACTGTG-3’		
TRE-Mi	5’-GTGAACCGTCAGATCGCCTG-3’	450	
	5’-CGGGTAACGTATTTGCCATTTG-3’		

Table S2. Primers and thermocycle parameters of RT-PCR for DMV mice. Thermocycle program: (1) 94°C, 5min. (2) 94°C, 0.5 min. (3) indicated temperature, 0.5 min. (4) 72°C, 1 min. Repeat (2)-(4) for the indicated cycle number. (5) 72°C, 10mi

Target	Forward and reverse primer	Size (bp)	Annealing temp. (°C)	Cycle number
Mi-V5 specific	5’-CCCGTCTCTGGAAACTTGATCG-3’	842 bp	57	30
	5’-ACCGAGGAGAGGGTTAGGGAT-3’			
Total Mitf	5’-CCCGTCTCTGGAAACTTGATCG-3’	818 bp	57	30
	5’-GCTCTCCGGCATGGTGCCGAGG-3’			
Mitf-M specific	5’-TGCTGGAAATGCTAGAATACAGT-3’	996 bp	57	30
	5’-GCCTGGTGCTGTACAAGTTC-3’			
Dct	5’-TTCCCCGAGTCTGCATGAC-3’	212 bp	57	28
	5’-TGCATGTCCGGTTGAAGAATTT-3’			
Tyr	5’-CAAAGGGGTGGATGACCGTG-3’	101 bp	55	30
	5’-AACTTACAGTTTCCGCAGTTGA-3’			
Best1	5’-AGGATGAGGAAGGCCGTTTG-3’	460 bp	57	30
	5’-TCCATCTCATGGCCTGGGTA-3’			
Rdh5	5’-CAGGCCAGGGGTCGGGTGGT-3’	500 bp	57	30
	5’-TCAGGAGACTGACTGGGCGG-3’			
Rlbp1	5’-ACAAGTATGGTCGAGTGGTT-3’	499 bp	57	30
	5’-TCATAAGGCTGTGTTCTCAA-3’			
Gapdh	5’-TGCACCACCAACTGCTTAG-3’	176 bp	57	30
